# Interactive teaching environment for diagnostic radiography with real-time X-ray simulation and patient positioning

**DOI:** 10.1007/s11548-021-02499-7

**Published:** 2021-10-13

**Authors:** Aaron Sujar, Graham Kelly, Marcos García, Franck P. Vidal

**Affiliations:** 1 Universidad Rey Juan Carlos, Madrid, Spain; 2 Bangor University, Bangor, UK; 3 Shrewsbury and Telford Hospital NHS Trust, Shrewsbury, UK

**Keywords:** Virtual reality, Medical simulation, X-rays, Diagnostic radiography, Medical diagnostic imaging, Training

## Abstract

**Purpose:**

Traditional undergraduate radiographer training mixes academic lectures and clinical practice. Our goal is to bridge the current disconnection between theory and practice in a safe environment, avoiding the risk of radiation for both practitioners and patients. To this end, this research proposes a new software to teach diagnostic radiography using real-time interactive X-ray simulation and patient positioning.

**Methods:**

The proposed medical simulator is composed of three main modules. A fast and accurate character animation technique is in charge of simulating the patient positioning phase and adapts their internal anatomy accordingly. gVirtualXRay is an open-source X-ray simulation library and generates the corresponding radiographs in real time. Finally, the courseware allows going through all the diagnostic radiology steps from the patient positioning and the machine configuration to the final image enhancing.

**Results:**

A face and content validation study has been conducted; 18 radiology professionals were recruited to evaluate our software using a questionnaire. The results show that our tool is realistic in many ways (72% of the participants agreed that the simulations are visually realistic), useful (67%) and suitable (78%) for teaching X-ray radiography.

**Conclusions:**

The proposed tool allows simulating the most relevant steps of the projectional radiography procedure. The virtual patient posing system and X-ray simulation module execute at interactive rates. These features enable the lectures to show their students the results of good and bad practices in a classroom environment, avoiding radiation risk.

## Introduction

Projectional radiography is a very common medical imaging tool that supports clinicians in the diagnosis of certain diseases, infections, injuries, to locate foreign objects, etc. It can be used on almost every part of the patient’s body, although each specific body location requires a given patient position and X-ray machine set up, in particular, collimation of the X-ray source, voltage of the X-ray tube, time of exposure, distance source to patient, and distance source to detector or film.

Undergraduate radiographer training within the UK is a mix of academic theory and clinical practice. This approach leads to a disconnect between the theory seen in the classroom setting and the practice seen in the X-ray room. Trainee radiographers learn theoretical information such as anatomy and radiation physics. Theoretical information is learnt within the classroom.

Many institutions rely on creative simulations in order for the trainee to experience the clinical environment within a non-clinical setting such as the university. Depending on the university facilities, trainees may use X-ray phantom anatomy to further their understanding of radiation physics principles without the risk of biological damage to living tissue.

Trainees can then see the effect of imaging principles, such as exposure factors, in real time, a practice that could not be performed on living tissue. Well-documented large data sets of cases are utilised within the academic setting again to demonstrate clinical practice. They are compilations of patient histories including their medical images, recorded discussions, notes and annotations. University hospitals and medical schools often build their repositories. However, there are now more and more online public resources that radiographers can access to consult vast sets of images from any part of the human anatomy [1]. Nowadays, digital technologies are more ubiquitous than ever, and smartphones start to play a big role in learning and teaching. Some institutions have created mobile phone apps in which trainees can look up images and fill in questionnaires, e.g. UBC Radiology [2]. These types of resources are playing an increasing role in the curriculum of trainees in radiology and diagnostic radiography. The use of mobile phone apps and classical teaching cases suffer, however, from the same limitations. They show the final results and do not describe the whole procedure. For safety reasons, projections obtained with different setups never come from the same patient, hindering any possible comparison. As they are static images, it is not possible to modify acquisition parameters (e.g. tube voltage) and interactively visualise the changes in the X-ray radiographs. Also, patient positioning is a key step, and it cannot be easily learned from the final images.

It is a vital aspect of undergraduate training that mistakes are learnt from to ensure they are not replicated within the real clinical setting. For trainees to further understand errors, an X-ray room is required within the academic setting. The use of an anatomical phantom is also required for X-ray to demonstrate these errors. This cannot be easily conducted within a typical classroom setting and therefore constitutes a separate teaching session, leading to a disconnect between theory and practice.

Virtual reality (VR) medical simulation is playing an increasing role in the physicians’ curriculum  [3]. They are applied to a wide variety of medical procedures [4]. Such applications are used for safe and effective training purposes. Unlike traditional methods (using cadavers, animals or mannequins), computerised simulators permit new physicians to improve and develop their non-cognitive skills in a cheap and safe environment [5]. It is also possible to use such simulators for self-directed training without the close supervision of an expert.

Recent advances in computer graphics (CG) allow the development of new simulators that can be used in the apprenticeship of radiographers. Villard et al. [6] presented a simulator for training in interventional radiology. Fluoroscopy (real-time X-ray images) was used to guide a needle towards an anatomical structure. This VR system implements interactive X-ray simulation and is able to animate the lung deformation. Nevertheless, it is not meant for teaching projection radiography, and it is constrained to the chest area. ProjectionVR^™^ [7] is a simulator that immerses the user into a realistic 3D X-ray room to simulate the complete procedure. Although it provides a variety of real cases, the patient positioning phase is limited, and no radiographs of the flexed articulations can be taken. Other simulators, such as MITE (Medical Imaging Training Immersive Environment)  [8] and CETSOL VR Clinic [9], are focused on training dynamic interaction and communication with the patient. They allow the trainees to go through all the procedure steps, including the patient positioning, but the final images are not physically based simulated.

Patient variability is desirable so that trainees can be exposed to all kinds of possibilities, e.g. from underweight to overweight patients, from babies to elderly patients. For this purpose, different virtual human models are required [10]. There are commercially available anatomical virtual human models, such as ZygoteBody^™^ [11]. An alternative is the use of real patient data captured using medical imaging techniques such as computed tomography (CT), magnetic resonance imaging (MRI) and/or ultrasound (US). The rationale is to provide more realistic models as there are directly derived from actual patient data [12]. The new generation of medical simulators has recently begun to use this kind of data [13].

For both commercial models and models generated from real data, virtual patients are often given in a specific position, different from that required in the medical procedure. Character animation techniques from CG (such as [14] can be used to move bones and update the skin surface accordingly. Unfortunately, they cannot transform the character’s internal anatomy. Biomechanical and muscle-skeletal techniques and physically based models offer accurate results  [15]. However, an expert must generally perform some steps manually and, in general, biomechanical models are not complete and localised only on a part of the human anatomy [16]. All these techniques do not run at interactive rates and may require detailed patient information, which is not readily available. As an alternative, Sújar et al. [17] proposed a fully automatic method to deform anatomical structures at interactive rates.

Another component that is required to build this interactive environment is the X-ray image simulator. Both accuracy and speed are requirements. Physically based simulation frameworks aim at particle physics and/or medical physics research and focus on accuracy rather than speed [18]. As a fast alternative, a deterministic calculation based on ray-tracing is often used to solve the Beer-Lambert law [19]. Freud et al. proposed an alternative model for deterministic simulation [20] that relies on the traditional graphics pipeline. It has been ported to modern graphics processing unit (GPU) using OpenGL [21], providing a real-time X-ray image simulation tool. It is now available as an Open-Source project, gVirtualXRay [22].^1^

This research proposes an X-ray projectional radiograph simulator to teach and train the procedure in a safe environment. Teachers and students can change the patient positioning and the X-ray machine parameters interactively and see the effects of their actions on the final image. We aimed to create a software that could be used in the classroom to help bridge the gap between theory and practice. Our tool was designed with the following requirements in mind:

- It can simulate the most important steps of the procedure safely in the classroom environment.
- It could use a wide variety of existing virtual patient models.
- Users can interactively manipulate the virtual patient to the position required in any given procedure.
- Users can observe the X-ray image immediately as they change the virtual patient’s position, X-ray source’s position or any other X-ray machine parameter.

A YouTube Playlist with videos was created to illustrate the most important functionalities of our interactive teaching and learning environment.^2^

## Methods

The proposed tool allows teachers and students to interact with the X-ray configuration and the patient’s positioning without any kind of radiological risk of the patient or even the radiographer. This approach relies on three main modules: The Virtual X-ray Imaging Library (gVirtualXRay), the Virtual Patient Positioning System (VPPS) and the Courseware. The first one is responsible for generating the X-ray image in real time, while VPPS transforms the anatomy of a virtual patient to the desired position. Finally, both modules are integrated into a courseware environment, which implements the user interface and characterises the most important steps in X-ray projectional radiography.

### Virtual patient positioning system

To let the user select a position in real time, this module adjusts the internal and external anatomical models of a virtual character to any desired position. It follows a purely geometrical approach based on an improved skeletal animation technique [17], instead of a fully physically-based simulation algorithm, to meet the following requirements:

- It has to be flexible to incorporate as many existing 3D patient models as possible.
- It has to minimise user intervention when adding new virtual patients.
- It has to run in real time.

Computer graphics skeletal animation workflow transfers the movement of a virtual skeleton to a boundary representation (B-rep) of a virtual character. The main limitation of this workflow is that some steps are usually performed manually by a 3D artist to provide a plausible animation. Although several approaches automatise these stages, they only work with B-reps, and they cannot be used to deform internal soft tissues. The proposed method relies on a fully automatic procedure that transfers the bone movements to all tissue models and is divided into five steps:

- Rigging: A predefined virtual skeleton is adapted to the virtual patient labelled bone tissue.
- Volumetrisation: This stage automatically builds a volumetric Lagrangian mesh from the patient skin and bones.
- Weighting: The influence of the virtual bones on the volumetric mesh vertices is calculated using the diffusion equation for the stationary case.
- Mapping: All the virtual tissues are mapped into the volumetric mesh to accelerate the real-time animation of the patient anatomy.
- Skinning: The virtual bones movements are applied to the volumetric mesh and then transferred to the patient tissues.

The module is implemented to run on modern GPUs and achieves interactive frame rates, even for complex models (see Sect. 3).

### Virtual X-ray imaging library

A deterministic simulation tool is required to generate images in real time. Monte Carlo simulation is not suitable as it cannot provide real-time performance. gVirtualXRay has been selected for the X-ray generation as it is an open-source library that makes use of portable technologies.

gVirtualXRay implements Freud et al.’s L-buffer principle [20] on the GPU. It was first implemented on GPU with a monochromatic beam spectrum (all the photons have the same energy) and for parallel projections and infinitesimally small point sources [21]. The current version of gVirtualXRay relies on the XCOM Photon Cross Sections Database from the National Institute of Standards and Technology (NIST) to compute the mass attenuation coefficient of the material of the scanned objects [23]. The material can be defined as a chemical element (e.g. hydrogen), a mixture (e.g. “Ti90Al6V4” for a titanium alloy with small amounts of aluminium, 6%, and vanadium, 4%), a compound (e.g. “H2O” for water), or a Hounsfield unit. The latter has been chosen as it is commonly understood by the medical community. Hounsfield units are then converted into their respective chemical compositions and densities [24]. A quantitative validation study has been conducted to assess the accuracy of the simulated X-ray images [22].

### Courseware

**Fig. 1:**
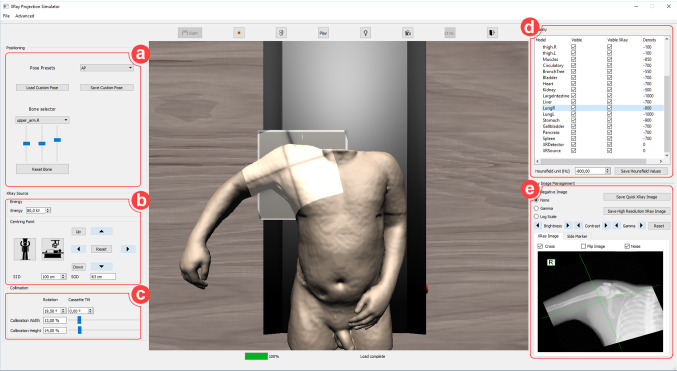
GUI of the projectional radiography simulator: **a** patient positioning options, **b** centring, tube voltage, source-object distance and source-cassette distance configuration **c** collimation setup, **d** tissue visibility and material properties, **e** side marker’s selection and digital image manipulation

The courseware is in charge of developing the teaching and learning environment, integrating the previously described modules and providing a graphical user interface (GUI). The previously described modules are both implemented on the GPU. In fact, both modules have been integrated in such a way that they share the virtual patient data on the GPU memory to increase the system speed. Figure 1 shows how the GUI looks like and the main functionalities of the simulator. The following sections explain how the system decomposed the procedure into different stages and the simulator’s lecturing and self-guided training functionalities.

#### Positioning the patient

In projectional radiography, the positioning of a patient is essential to get the best image whilst reducing the radiation dose to the minimum requirement. As described in the radiographer curriculum [25], the users can choose if the patient is standing up or lying down. Note that the VPPS makes use of a purely geometrical algorithm and does not take into account the effect of gravity. Users can define the position of the patient using 3 methods: i) Common positions used in radiography can be selected from a list; ii) They can eventually choose any body part and move it directly; iii) They can use motion capture devices such as Microsoft Kinect.^3^ The 3D model used for Fig. 1 is from the Visible Human data set, i.e. taken from a medical CT scanner where the arms are alongside the body. In Fig. 1, the upper arm was moved to get an AP humerus projection. Any position can be saved so that teachers can make pre-recorded positions available to students or for future use in the classroom.^4^

#### X-ray setup

**Fig. 2:**
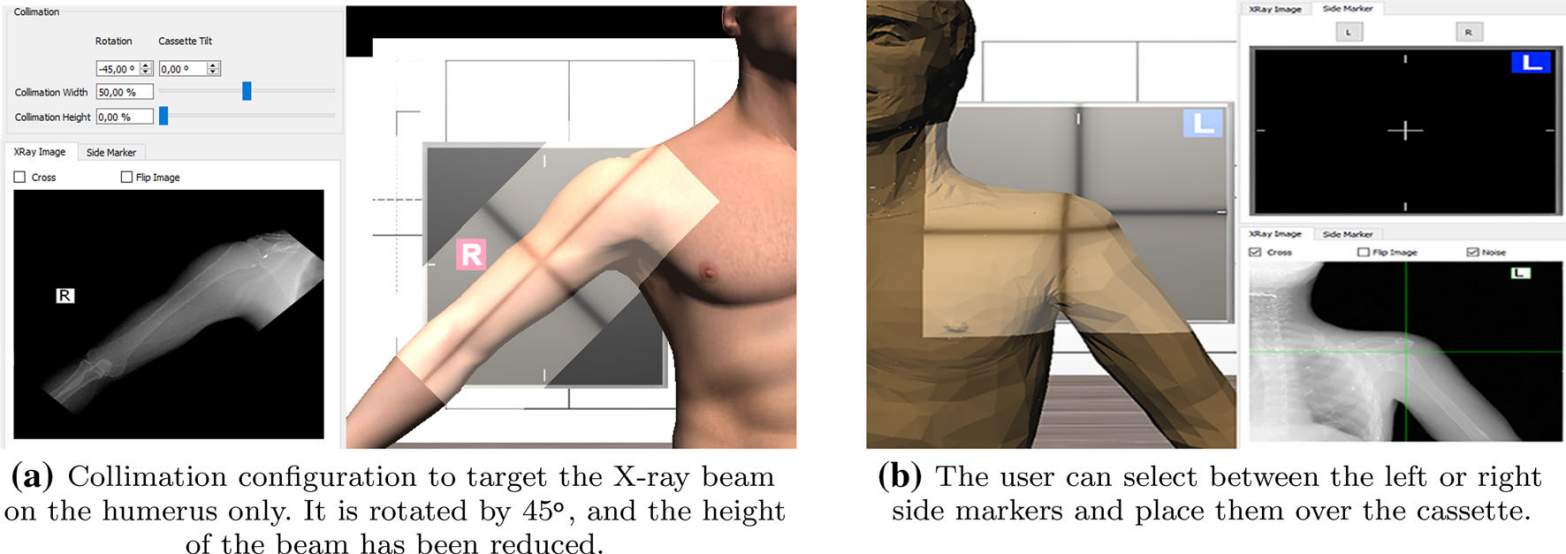
Users can move and configure the cassette to get an X-ray image

**Fig. 3:**
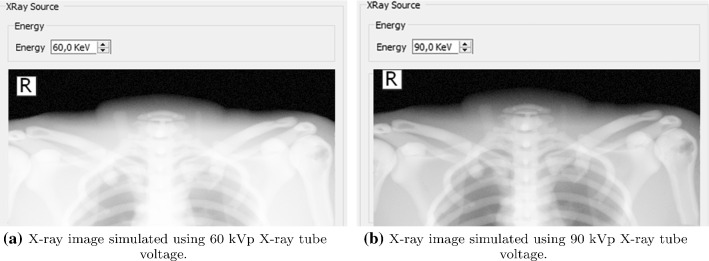
Effect of the X-ray tube voltage on an X-ray image of the chest

After patient positioning, the X-ray source and detector (cassette) need to be configured. During the *centring*, the primary X-ray beam focus is placed in relation to the anatomy and X-ray detector. Additionally, the cassette must be placed and aligned. These steps and patient positioning are heavily correlated. They are crucial to maintain the radiation dose to its strict necessary minimum and have a direct impact on the final image quality. In the proposed system, users need to define the following parameters (Figs. 2a and 3): X-ray beam focus position, source to image distance (SID), source to object distance (SOD), cassette position. Lead plates or leaves can be placed at the front of the X-ray tube to limit the exposure to ionising radiation to a given area of the body (*Collimation*). Appropriate collimation is also important to ensure the area of interest is included in the image, reducing the radiation field to that area of interest and improving the image quality lowering the noise. Users can perform the collimation by configuring the width, height and orientation of the beam (Fig. 2a).

In clinical radiography, a marker is placed within the field of the X-ray source (clear of the area of interest) to identify the left and right-hand sides of the patient. This practice is mandatory to avoid misinterpretation of the X-ray image. In this tool, a 2D widget allows placing a side marker (Fig. 2b) over the cassette using the mouse.^5^ interactively.

The X-ray beam spectrum corresponds to a tabulated list of a number of photons and corresponding photon energies in kiloelectron volt (keV). In a clinical environment, this is controlled by radiographers via the keV of the current applied to the X-ray tube. Exposure factors play an important role in X-ray projectional diagnosis because they can affect the quality of the produced radiograph. An inappropriate exposure may decrease the contrast in X-ray images. To avoid mistakes and repetitions, trainees must understand how radiation is produced by an X-ray tube and what/how parameters affect the image quality. In our system, the user can select the voltage in kilovolt(kV), and gVirtualXRay uses the corresponding beam spectrum (see Fig. 3). If the kV value is too high, the image will be over-exposed and too dark. In contrast, low kV generates whiter and blurred images.

#### Digital image manipulation

Digital X-ray detectors are commonly used in modern hospitals rather than traditional X-ray films. The greatest advantage of digital imaging is the ability to distribute, store and manipulate the data. Image filtering can be used to reduce problems such as overexposure or underexposure. The system user interface provides access to a few simple image processing techniques that allow users to enhance resulting images.^6^ that allow users to enhance resulting images: Log-Scale filtering, Gamma filtering, contrast and brightness adjustment, and *negative* image.

#### Lecturing features and self-guided training

The proposed simulator provides additional features which will support the instructor in the classroom environment, although they are not directly related to the simulation of any step of the diagnostic radiography procedure. To reveal internal structures in the 3D visualisation window, the system allows to show, hide or modify the opacity of specific tissues at run time.

**Fig. 4:**
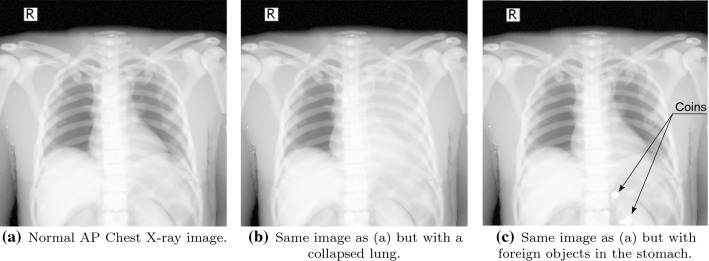
X-Ray attenuation properties of the tissues can be modified to simulate different scenarios

It is also possible to modify tissues’ attenuation properties using the Hounsfield scale, being able to show a variety of diseases, e.g. a calcified bone, a collapsed lung (Fig. 4b), an air-filled stomach. It is even possible to introduce foreign objects inside the internal anatomy.^7^ (Fig. 4c).

The only effective way of acquiring non-cognitive skills is practice. VR simulators can be exploited to boost the trainees’ learning process in a self-directed manner. We offer a set of non-guided exercises that allows the instructor to check the evolution of the trainees. A set of videos were created to show these features and help the user.^8^

## Results

### Face and content validation

A combined face and content validation study has been conducted to gather feedback from experts. Ethical approval for this experiment was obtained (see details below), and participants’ written informed consent was collected before their enrolment in the study.

The face validation evaluates the level of resemblance between the simulation and the procedure performed in the real world. Content validation quantifies what the tool actually teaches/trains (e.g. psycho-motor skills or anatomy). It ascertains that the simulation correctly replicates the steps and features of the real procedure. The distributed form includes questions designed: i) to characterise the cohort of participants and assess their level of expertise in X-ray radiography, ii) for the face validation, and iii) for the content validation.^9^

A call for participation was distributed among radiography and radiology professionals. Eighteen volunteers answered the call. The volunteers were not filtered. None of the participants was involved in the project or had used or seen the tool previously. The average number of years of experience is 12.5 years (standard deviation 11.74 years). This cohort is well experienced to assess our tool. There were 9 males and 9 females. It allows us to check if there is any gender effect. All the participants but one are qualified. Although most participants were from the UK (15 of them), 1 participant was from Canada, 1 from France and 1 from Spain; 16 participants exercise their art in radiography, 1 participant is a medical doctor (MD) in stomatology and another one in nuclear medicine. Due to his speciality, the stomatologist reported being confident when taking X-ray radiographs. However, the MD in nuclear medicine did not. Both MDs reported being extremely confident when interpreting X-ray radiographs. As a consequence, their answers were relevant to our study.

**Fig. 5:**
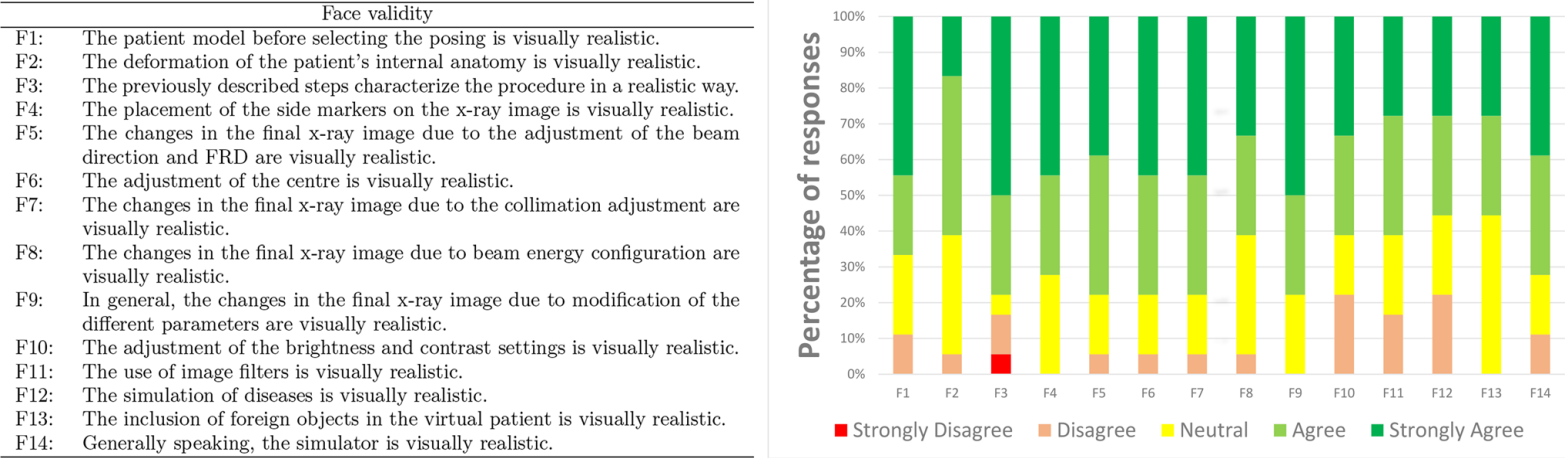
Result of the face validation study. Participants were asked to express their degree of agreement with each of the statements using a Likert scale

Participants were asked to rate to what extent they agreed on each statement. They were told to use a five-point Likert scale from 1 (strongly disagree) to 5 (strongly agree); 14 statements related to the realism of the simulator and its functionalities were used for face validity. Figure 5 shows the corresponding questions and results for all participants.

The percentage of ‘strongly agree’ or ‘agree’ answers for each statement is above 50%. Between 70% and 79% of the participants ‘strongly agree’ or ‘agree’ with seven of the statements (F3, F4, F5, F6, F7, F9, and F14). It is between 60% and 69% for five statements (F1, F2, F8, F10, and F11). It is between 50% and 59% for only two statements (F12 and F13), which are related to the mimicking of disease and the inclusion of foreign objects. This functionality is in its preliminary stage. It was implemented using a relatively naive approach (e.g. changing the HU value for the collapsed lung). The overall feedback is positive (F14). This initial face validation is promising as it clearly shows that most participants ‘strongly agree’ or ‘agree’ that the simulator is realistic in many ways.

**Fig. 6:**
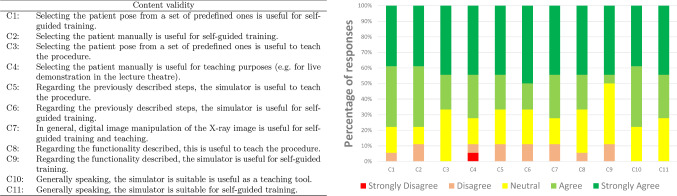
Result of the content validation study. Participants were asked to express their degree of agreement with each of the statements using a Likert scale

For content validity, 11 statements related to the main purpose of the simulator and the courseware were included. Figure 6 shows the corresponding questions and results for all participants.

A pattern similar to the face validation is observed for the content validation. Between 70% and 79% of the participants ‘strongly agree’ or ‘agree’ with six of the statements (C1, C2, C4, C7, C10, and C11). It is between 60% and 69% for four of the statements (C3, C5, C6, and C8). It is 50% for C9. The lower score at F12 and F13 did not affect the content validation as the main goal of the simulator is not to teach image interpretation. In fact, the overall feedback is positive. Participants judged that the tool is useful as a teaching tool for X-ray radiography (C8), but less as a self-guided training tool (C9). However, the results show the suitability of the proposed tool as a teaching tool (C10) and as a self-guided training tool (C11).

Participants also had the opportunity to provide free comments. When provided such comments were very positive: “I think this would be a really excellent tool for students to learn from and improve their skills for when they go in placement.” and “Great idea”.

### Performance results

The proposed X-Ray simulator offers a teaching and learning platform where third party anatomic models can be easily integrated. To test the viability of this approach, the following models were tested:

- *ZygoteBody*^™^ 3D Poly Models [11]. This set of virtual patient models provide B-Reps of the most important tissues. We used female (ZF) and male (ZM) models.
- *Anatomium*^™^ 3D Human Anatomy Digital Data Sets offer several virtual patients. Similarly to ZygoteBody, they include the B-reps of several tissues. We used one of the available male models.
- *Voxel-Man’s* Segmented Inner Organs [26]. This model is composed of a set of segmented volumetric images obtained from the Visible Human data set  [27].

**Fig. 7:**
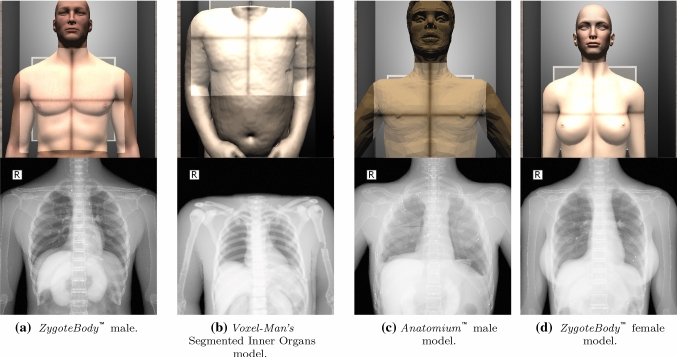
Results obtained using different anatomical models

**Fig. 8:**
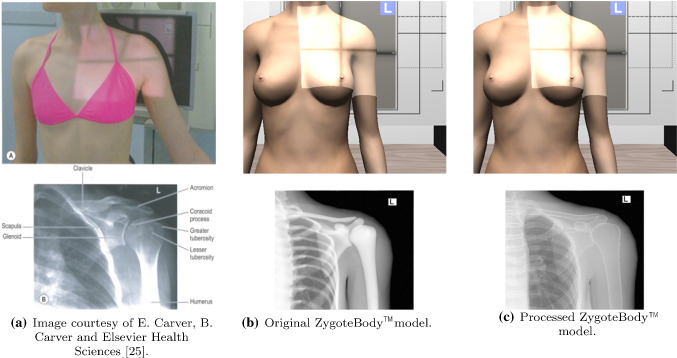
Proposed system can replicate any type of projection, allowing the user to select the patient position and the X-ray configuration

Figure 7 depicts the visual results obtained for these models. As expected, the quality of the final image is highly dependent on the quality of the virtual patient model. Some general-purpose models need further pre-processing to obtain plausible images. Commercial 3D virtual models often only provide the bone structures as the surface of the cortical bones (see Fig.8b). To solve this deficiency, a new mesh for each bone was added to depict the trabecular bones (see Fig. 8c). Note that this issue does not occur when working with surface meshes extracted from segmented CT data set (see Fig.7b) as cortical and trabecular bones are easy to identify and separate in CT data.

## Conclusions

In this paper, a VR teaching and learning environment for diagnostic radiography was presented. It relies on i) an interactive character animation method for the patient positioning and deformation of internal tissues, and ii) a real-time X-ray simulation library. The courseware module turns the simulator into a teaching tool.

Radiographers have to know both how to position a patient and how to tune an X-ray tube to avoid clinically unnecessary radiation doses and repetitive acquisitions of X-ray images. The proposed system provides a safe environment where the procedure can be taught and rehearsed. Thanks to the computational efficiency of the VPPS and gVirtualXRay, lectures can interactively show to their students the results of good and bad practices in a classroom environment. Furthermore, this solution was designed to facilitate the incorporation of new virtual patient models to provide anatomical variability. This functionality was particularly challenging since it allows the user to modify the patient’s position interactively, and very few constraints are imposed on the models.

Although the presented simulator is a standalone application, the two individual components, the VPPS and the Virtual X-ray Imaging Library, can be incorporated into other VR medical applications. VR patient data sets can be built from real patient data. Unfortunately, medical imaging techniques capture patient data in specific positions. In most cases, our posing system is flexible and robust enough to adapt the patient position to the one required by the procedure. Our X-ray simulator can be incorporated to simulate any medical technique that involves X-ray imaging. For example, in the context of cardiovascular interventions, VCsim3[4] allows the user to visualise the virtual catheter/guidewire pair using X-rays. Their heuristic and no-accurate X-ray module can be substitute by ours, enhancing the user experience.

Nevertheless, this tool has some limitations. The positioning system scarifies accuracy in favour of computational performance and flexibility. For example, it ignores gravity. Additionally, the X-ray simulation algorithm cannot fully consider mAs effects due to its deterministic nature. In this algorithm, all photons reach the detector following the same path. Scattering and photon noise would require implementing a stochastic simulation. However, it is possible to mimic a low mAs value by adding Poisson noise to the simulated X-ray image. The amount of noise can be calibrated based on real images using different mAs and kVp. Finally, patient preparation is not covered in this tool: patient communication, pregnancy protocols, etc.

As future work, the potential of the simulator will be assessed. A user study with undergraduate radiographers will be conducted to test if the skills acquired in the context of the simulator are successfully transferred to the real world. Then, the self-directed learning capabilities of the system will be increased. These new functionalities would require the design and implementation of new courseware content and features and their validation. Computerised systems are good at registering user actions. All this information can be used to develop a set of assessment metrics that will serve to provide the trainees with information to boost their self-learning processes. Additionally, these metrics can be used to evaluate their proficiency. The development of a proper set of assessment metrics is not trivial and requires further research and rigorous validation.

## Footnotes

1 http://gvirtualxray.sourceforge.net/.

2 https://www.youtube.com/playlist?list=PLI54dBkP-i2QjkHx10Z6FCmlDW_kZUAIX.

3 https://youtu.be/1s-eTT1pCy8.

4 https://youtu.be/zitw5-Xk4dY.

5 https://youtu.be/EDJM3pSWtgo.

6 https://youtu.be/x7KGsNSMSh8.

7 https://youtu.be/O2K2ojcExTc.

8 https://youtu.be/GwWY8AQffEY and https://youtu.be/WFOAVSLXufs.

9 The questionnaire is available at https://goo.gl/forms/HyEIQwedhF8m9XuS2.

## References

[1] Deshpande P, Rasin A, Brown E, Furst J, Raicu D, Montner S, Armato S III (2017) An integrated database and smart search tool for medical knowledge extraction from radiology teaching files. In: Proceedings of workshop on medical informatics and healthcare 10–18

[2] Spouge R (2017). Review of UBC Radiology Teaching App. J Dig Imag.

[3] Bernardo A (2017). Virtual reality and simulation in neurosurgical training. World Neurosurg.

[4] Korzeniowski P, White RJ, Bello F (2018). VCSim3: a VR simulator for cardiovascular interventions. Int J Comput Assist Radiol Surg.

[5] Patel R, Dennick R (2017). Simulation based teaching in interventional radiology training: is it effective?. Clin Radiol.

[6] Villard PF, Vidal FP, Ap Cenydd L, Holbrey R, Pisharody S, Johnson S, Bulpitt A, John NW, Bello F, Gould D (2014). Interventional radiology virtual simulator for liver biopsy. Int J Comput Assist Radiol Surg.

[7] Shanahan M (2016). Student perspective on using a virtual radiography simulation. Radiography.

[8] Bridge P, Gunn T, Kastanis L, Pack D, Rowntree P, Starkey D, Mahoney G, Berry C, Braithwaite V, Wilson-Stewart K (2014). The development and evaluation of a medical imaging training immersive environment. J Med Radiat Sci.

[9] Sapkaroski D, Baird M, McInerney J, Dimmock MR (2018). The implementation of a haptic feedback virtual reality simulation clinic with dynamic patient interaction and communication for medical imaging students. J Med Radiat Sci.

[10] Preim B, Saalfeld P (2018). A survey of virtual human anatomy education systems. Comput Graph.

[11] Zygote Media Group. ZygoteBody. [Online]. Available: https://www.zygotebody.com/ (2018). Accessed: 2018-11-08

[12] de Oliveira JE, Giessler P, Keszei A, Herrler A, Deserno TM (2016) Surface mesh to voxel data registration for patient-specific anatomical modeling. In: Medical imaging 2016: image-guided procedures, robotic interventions, and modeling, vol. 9786 (International Society for Optics and Photonics, 2016), vol 9786, p 978625. 10.1117/12.2217491

[13] Zhang J, Chang J, Yang X, Zhang JJ (2017) In: Next Generation Computer Animation Techniques, Lecture Notes in Computer Science, vol 10582, ed. by J Chang, JJ Zhang, N Magnenat Thalmann, SM Hu, R Tong, W Wang (Springer, Cham), Lecture Notes in Computer Science, vol 10582, pp 220–233. 10.1007/978-3-319-69487-0_16

[14] Le BH, Hodgins JK (2016) Real-time skeletal skinning with optimized centers of rotation. ACM Trans Graph **35**(4), 37:1. 10.1145/2897824.2925959

[15] Rajagopal A, Dembia CL, DeMers MS, Delp DD, Hicks JL, Delp SL (2016). Full-body musculoskeletal model for muscle-driven simulation of human gait. IEEE Trans Biomed Eng.

[16] Ichim AE, Kadleček P, Kavan L, Pauly M (2017) Synthesizing obama: learning lip sync from audio. ACM Trans Graph **36**(4), 153:1. 10.1145/3072959.3073664

[17] Sújar A, Casafranca JJ, Serrurier A, García M (2018) Real-time animation of human characters’ anatomy. Comput Graph 74:268. 10.1016/j.cag.2018.05.025

[18] Baró J, Sempau J, Fernández-Varea JM, Salvat F (1995). PENELOPE: an algorithm for Monte Carlo simulation of the penetration and energy loss of electrons and positrons in matter. Nucl Instrum Methods Phys Res B.

[19] Glière A (1998) Sindbad: from CAD, model to synthetic radiographs. In Review of Progress in Quantitative Nondestructive Evaluation: Volume 17A (Springer, US. Boston. MA 387–394. 10.1007/978-1-4615-5339-7_49

[20] Freud N, Duvauchelle P, Létang JM, Babot D (2006). Fast and robust ray casting algorithms for virtual X-ray imaging. Nucl Instrum Methods Phys Res B.

[21] Vidal FP, Garnier M, Freud N, Létang JM, John NW (2009) Simulation of X-ray attenuation on the GPU. In: Theory and Practice of Computer Graphics, ed. by W Tang, J Collomosse (The Eurographics Association, 2009), pp 25–32. 10.2312/LocalChapterEvents/TPCG/TPCG09/025-032

[22] Vidal FP, Villard PF (2016) Development and validation of real-time simulation of X-ray imaging with respiratory motion. Comput Med Imag Graph 49:1. 10.1016/j.compmedimag.2015.12.002 10.1016/j.compmedimag.2015.12.002 26773644

[23] Berger MJ, Hubbell JH, Seltzer SM, Chang J, Coursey JS, Sukumar R, Zucker DS, Olsen K (2010) XCOM: Photon cross section database. Tech. Rep. NBSIR 87-3597, National Institute of Standards and Technology, Gaithersburg, MD. 10.18434/T48G6X. http://physics.nist.gov/xcom

[24] Schneider W, Bortfeld T, Schlegel W (2000) Correlation between CT numbers and tissue parameters needed for Monte Carlo simulations of clinical dose distributions. Phys Med Biol 45(2):459. 10.1088/0031-9155/45/2/314 10.1088/0031-9155/45/2/314 10701515

[25] Carver E, Carver B (2012). Medical imaging: techniques, reflection and evaluation.

[26] Segmented Inner Organs (SIO) Group. Voxel-Man. [Online]. Available: https://www.voxel-man.com/segmented-inner-organs-of-the-visible-human/. Accessed: 2018-11-08

[27] Lorensen WE, Cline HE (1987). SIGGRAPH. Comput Graph.

